# Abdominal Fistula: A Rare Postoperative Complication of Anterior Iliac Crest Graft Harvest in Bimaxillary Reconstruction

**DOI:** 10.7759/cureus.88471

**Published:** 2025-07-21

**Authors:** Mohamed Gafar Ahmed, Badr Al-Jandan, Meshall Almintakh, Mohammed K Bajunaid, Ahlam AlAbdali

**Affiliations:** 1 Department of Oral and Maxillofacial Surgery, King Fahd Hospital of the University, Dammam, SAU; 2 Department of Biomedical Dental Sciences, College of Dentistry, Imam Abdulrahman Bin Faisal University, Dammam, SAU; 3 Department of Oral and Maxillofacial Surgery, Imam Abdulrahman Bin Faisal University, Dammam, SAU; 4 College of Dentistry, Imam Abdulrahman Bin Faisal University, Dammam, SAU

**Keywords:** abdominal fistula, anterior iliac crest, asis bone grafting, enterocutaneous fistula, fistulectomy, iliac crest, posterior iliac crest

## Abstract

Autogenous bone grafts are considered the gold standard for reconstructing maxillofacial bony defects due to their osteogenic, osteoconductive, and osteoinductive properties. The anterior and posterior iliac crests are commonly used donor sites, providing sufficient quantities of bone for various clinical settings. However, iliac crest bone harvesting can lead to considerable postoperative complications such as wound infection, hematoma, pain, and gait disturbance. This report details a rare postoperative complication encountered by the Oral and Maxillofacial Surgery (OMFS) team at King Fahad University Hospital (KFUH): the formation of an abdominal fistula following anterior iliac crest bone harvesting for bimaxillary reconstruction. A 51-year-old female patient presented with a three-month history of an open abdominal wound with clear liquid discharge at the site of a previous left anterior iliac crest graft. Clinical and radiographic investigations, including a contrasted abdominal and pelvic CT scan, confirmed a deep abdominal cutaneous fistula extending into the left iliacus muscle. Fistulectomy was performed, and the patient showed satisfactory healing over a six-month follow-up period without recurrence. This case highlights the importance of thorough preoperative evaluation, awareness of potential complications, and consideration of alternative donor sites in the presence of previous scarring to minimize postoperative morbidity.

## Introduction

Autogenous bone grafts are the gold standard for reconstructing maxillofacial bony defects since the harvested bone possesses excellent osteogenic, osteoconductive, and osteoinductive properties [1]. Osteogenesis is defined as the process of new bone formation where osteoprogenitor cells in the graft material proliferate and differentiate into osteoblasts. The process of osteoinduction involves a biological response in which chemical signals induce osteogenesis. Osteoconduction is a physical, three-dimensional scaffold or matrix to facilitate bone repair [2].

Various donor sites, such as the tibia, anterior iliac crest, posterior iliac crest (PIC), costochondral, cranium, symphysis, and ramus, can be used for harvesting autogenous bone grafts [3]. The anterior and PICs are extremely adaptable grafts for maxillary and mandibular reconstruction. They are considered the most common donor sites that can provide a sufficient quantity of cancellous, cortical, or cortico-cancellous bone grafts to be utilized in different clinical settings. In addition, the preparation of the recipient site in the head and neck and the harvesting of bone can be done simultaneously when using the anterior iliac crest [4]. The anterior iliac crest graft (AICG) was first adopted by Chubb in 1920. It was performed for facial reconstruction in 60 cases of mandibular fractures [5]. The anterior iliac crest is a well-established donor site and can give up to 50 cc of noncompressed bone. In addition, it is more accessible than the PIC as a donor region, and bicortical grafts can be obtained [6].

On the other hand, iliac crest bone harvesting has some considerable postoperative morbidity and can result in significant postoperative complications such as wound infection, peritonitis, prolonged wound drainage, hematoma, prolonged postoperative pain, sensory changes, scars, gait disturbance, herniation of abdominal muscles and contents, and fracture of the iliac crest or pelvis [7,8].

The Oral and Maxillofacial Surgery (OMFS) team at King Fahad University Hospital (KFUH) encountered a rare postoperative complication of anterior iliac crest bone harvesting, which is abdominal fistula formation. A fistula is an abnormal connection or passageway between two organs or vessels that do not usually communicate. It can be classified according to etiology, organ of origin, etc. [9]. The development of a fistula is an uncommon yet significant complication following anterior iliac crest bone graft harvesting. Moreover, a review of the existing literature revealed no studies that specifically examine the risk of fistula formation as a postoperative outcome of this procedure.

## Case presentation

A 51-year-old female patient presented with a three-month-old abdominal wound complaining of mild pain and discomfort during movement associated with clear liquid discharge. She had a history of autogenous bone grafting from the left anterior iliac crest for bimaxillary reconstruction 10 months ago, which was done in the private sector. The patient was followed up by the private practice team for two months, but there was no improvement. The patient was then referred to KFUH for further evaluation and management. The reason for augmentation was to prepare the site before future implant placement. The patient gave no contributory medical history and no history of allergy to any medication with a healthy familial background. The patient underwent an abdominoplasty, a gastric sleeve, and a cesarean section four years ago.

**Figure 1:**
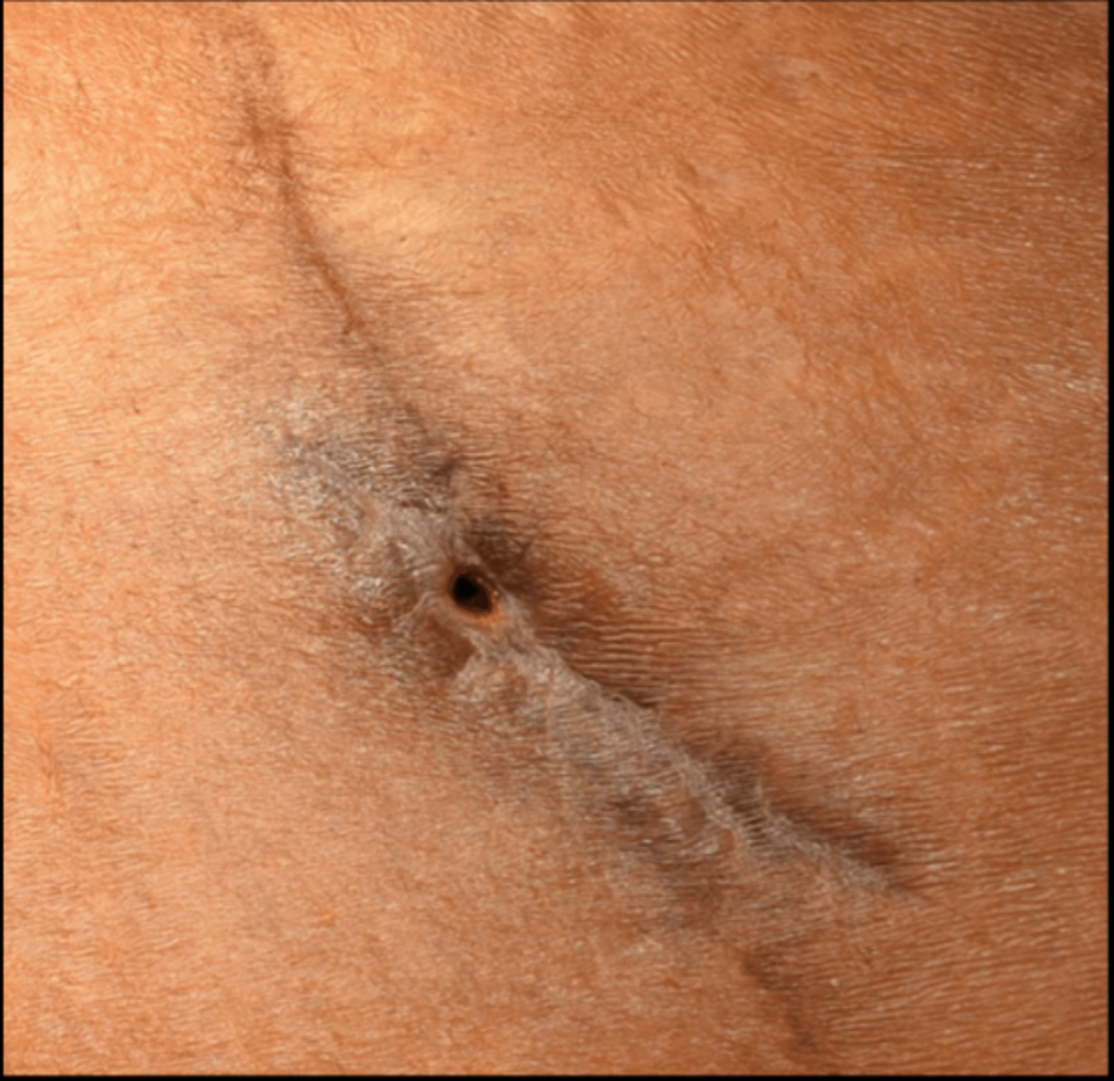
Appearance during the first visit shows an opening along the inferior margin of the iliac bone graft incision site.

Clinical examination at KFUH revealed scar tissue in the region of previous abdominoplasty and a scar from previous surgery at the site of bone harvesting 2 cm lateral and superior to the left anterior iliac crest and about 5 cm in length. The surrounding skin appeared to be normal. A small 5-6 mm abdominal fistula was seen in the middle of the healed scar of AICG (Figure 1).

Upon palpation, thin and discolored discharge from the abdominal fistula was induced with pressure. The surrounding linear scar is completely healed. A wound culture was obtained to rule out wound infection. The culture swab result was normal.

**Figure 2:**
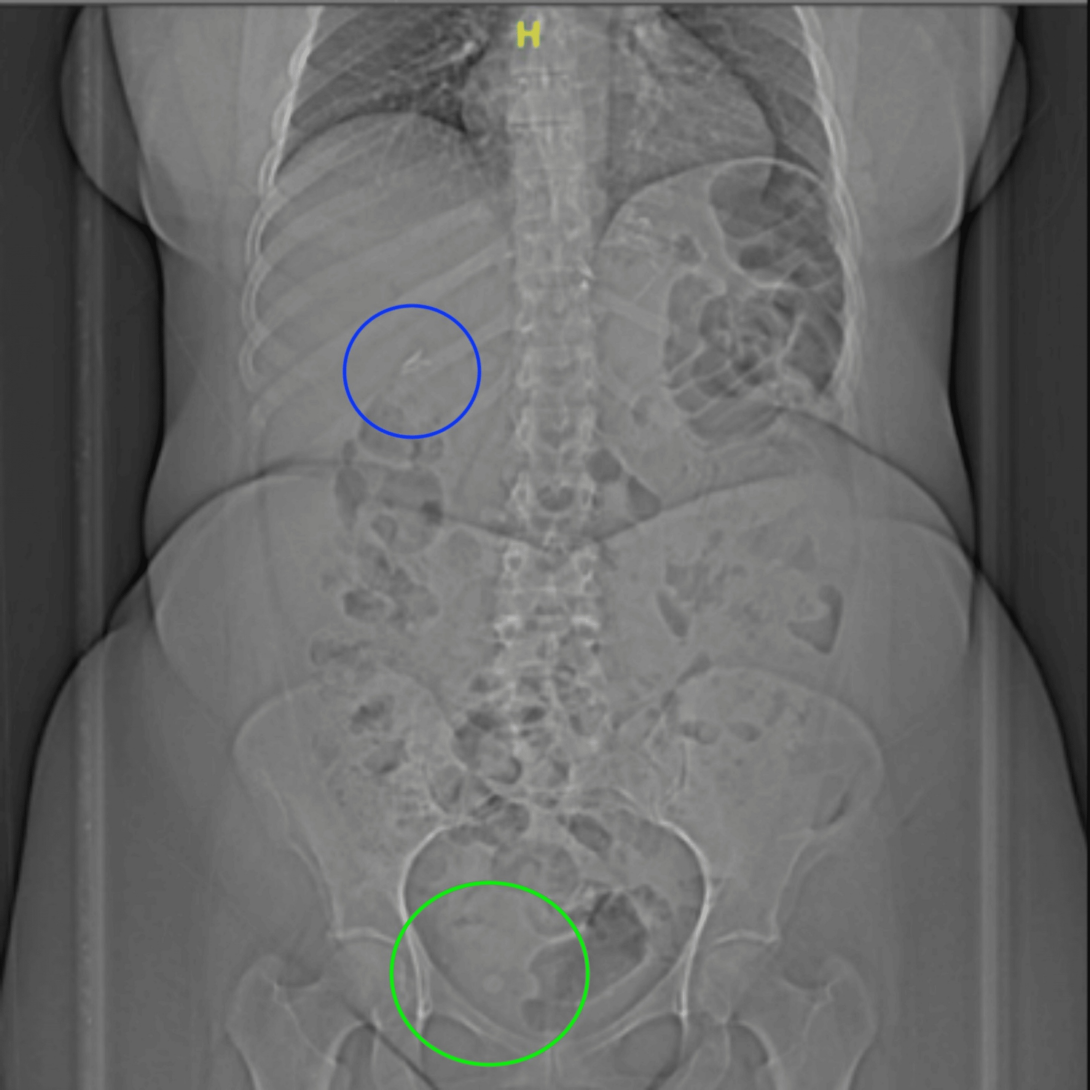
KUB X-ray revealed multiple surgical clips in the mid-abdomen and right upper quadrant (blue circle). Rounded radiopaque structures were noted in the pelvis, likely representing phleboliths (green circle). KUB, kidney, ureter, and bladder

A kidney, ureter, and bladder (KUB) X-ray was performed on the patient, which revealed multiple surgical clips at the mid-abdomen and right upper quadrant, with no definite calcific density noted in both renal shadows. Rounded radiopaque structures were seen within the pelvis, likely representing phleboliths. The bone was unremarkable (Figure 2).

**Figure 3:**
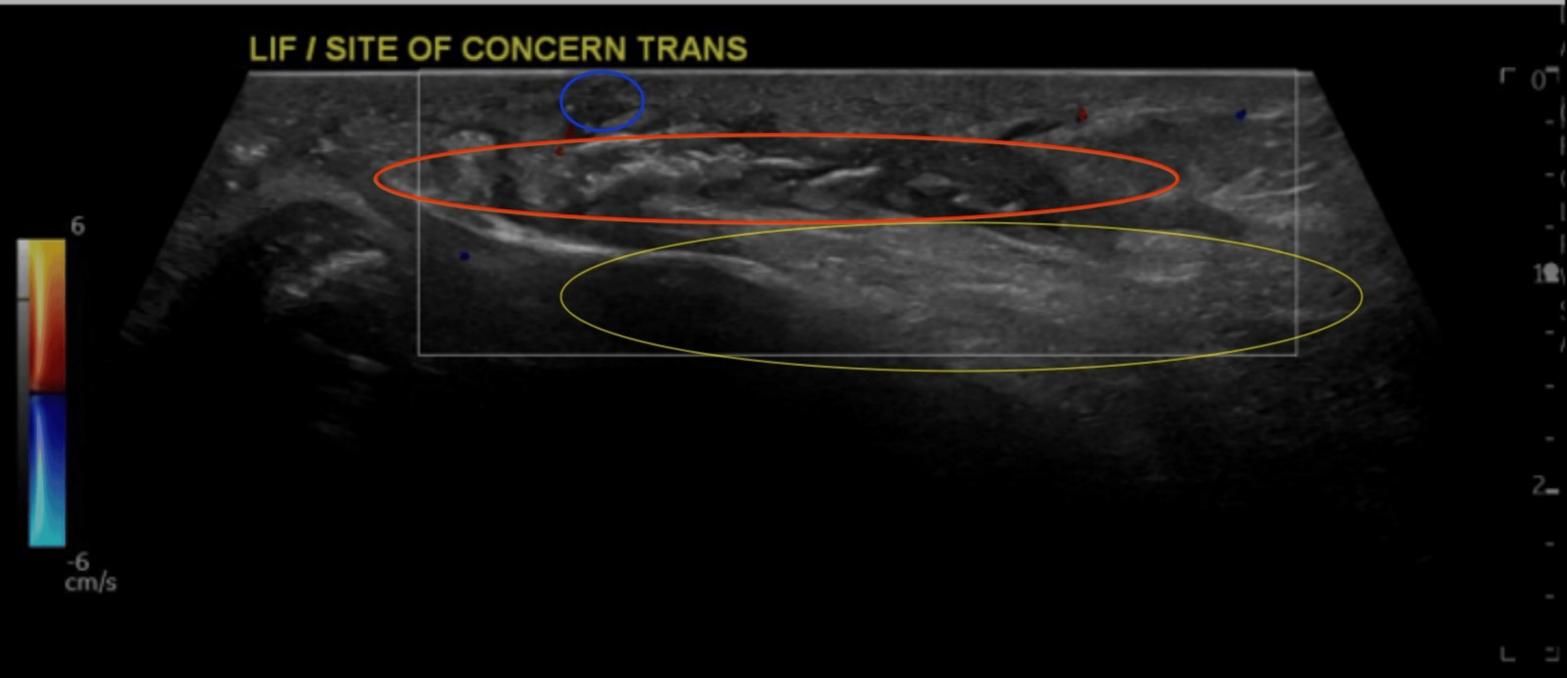
Ultrasound of the left hip surgical site revealed focal minimal superficial edema (yellow circle) associated with a fluid collection (red circle) tracking through a fine tract (blue circle) to the skin surface, without evidence of significant hypervascularity.

Ultrasonographic examination was performed and revealed evidence of focal edema on the left iliacus muscle at the level of the iliac bone, a donor site associated with a sub-centimetric pocket of fluid collection tracking deep through a fine tract to reach the skin surface without evidence of significant hypervascularity (Figure 3).

**Figure 4:**
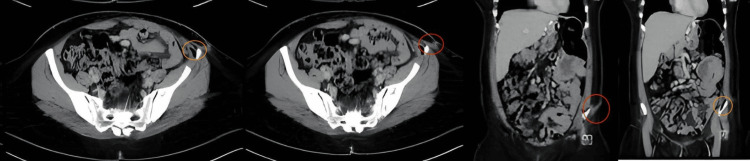
Axial and coronal CT images of the pelvis and abdomen reveal a tract extending from the subcutaneous tissue and skin into the left iliacus muscle at the level of the iliac bone (red circle). A small air locule is seen at the donor site, interposed between the iliacus muscle and iliac bone, without extension into the abdominal or pelvic cavity (orange circle). CT, computed tomography

Additionally, a contrasted abdominal and pelvic computed tomography (CT) scan was conducted to rule out the presence of an enterocutaneous fistula and to assess the depth of the identified fistula. (Figure 4)

The CT scan revealed a tract extending from the subcutaneous tissue about 7 cm in depth into the left iliacus muscle and at the level of the iliac bone donor site. A small air locule is seen interposed between the iliacus muscle and the iliac bone. However, there is no drainable collection and no extension into the abdominal, pelvic cavity, or viscera. 

**Figure 5:**
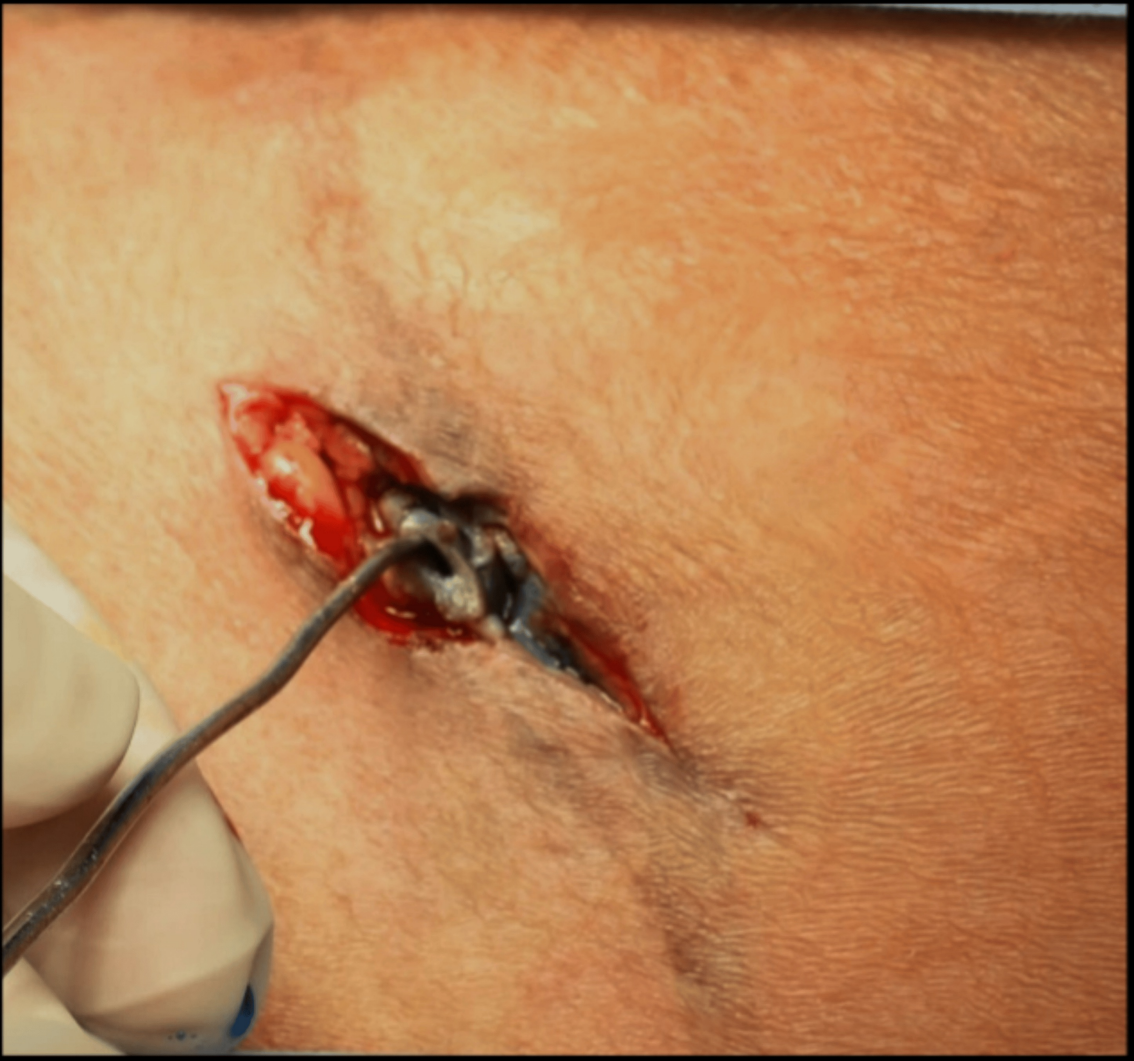
Skin incision and fistula tracing.

Following comprehensive clinical and radiographic evaluation, the OMFS team elected to perform a fistulectomy of the left deep abdominal cutaneous fistula under general anesthesia. In the operating room, the patient was placed in a supine position. General anesthesia was induced via oropharyngeal intubation, followed by skin preparation using povidone-iodine solution and sterile draping. An elliptical incision of 1 cm diameter was done around the fistula opening, which included the scar tissue. Methylene blue dye was injected into the fistula opening using a 26-gauge needle to identify the tract. Then, the fistula tract was traced using a fistula probe (Figure 5).

**Figure 6:**
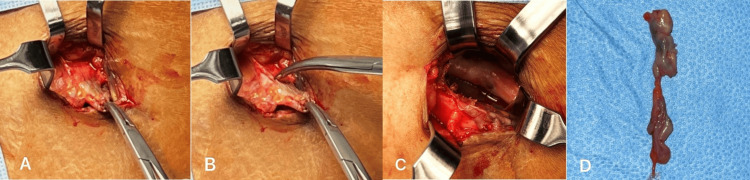
Intraoperative photographs showing: (A-B) dissection of the fistula tract; (C) identification of the anterior iliac crest and iliacus muscle; and (D) complete excision of the fistula for biopsy.

The skin was retracted using downward-curved surgical retractors. The subcutaneous tissue was dissected until the dye-containing fistula tract was visible. The fistula was held carefully with curved mosquito forceps, subcutaneous tissue was bluntly dissected, and the anterior iliac crest and iliacus muscle were identified. An excisional biopsy of the entire fistula tract was performed (Figure 6).

**Figure 7:**
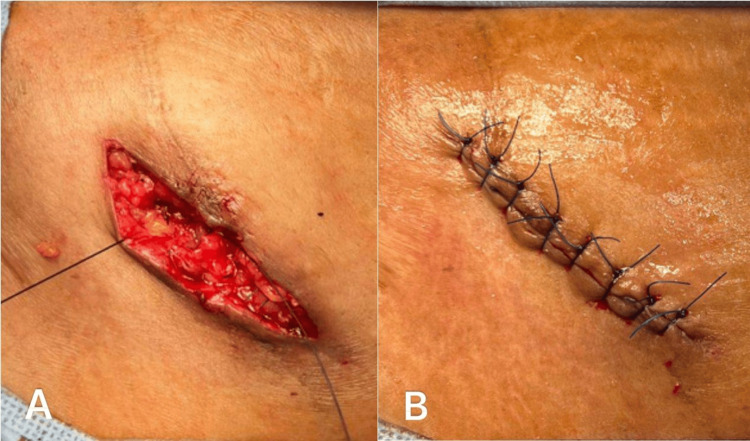
Suturing: (A) closure of deep muscle and subcutaneous layers in multiple layers using 3-0 Vicryl; (B) skin closure using 5-0 Prolene.

An approximately 6 mm-thick, elongated, irregularly tunneled tissue specimen measuring 70 mm in length was placed in 10% formalin in a labeled container for histopathological examination. Irrigation with normal saline (0.9% sodium chloride) was performed, and hemostasis was achieved. Closure was done in multiple layers using interrupted 3/0 Vicryl sutures and interrupted 5/0 Prolene sutures for the skin layer, followed by a tight pressure dressing (Figure 7).

**Figure 8:**
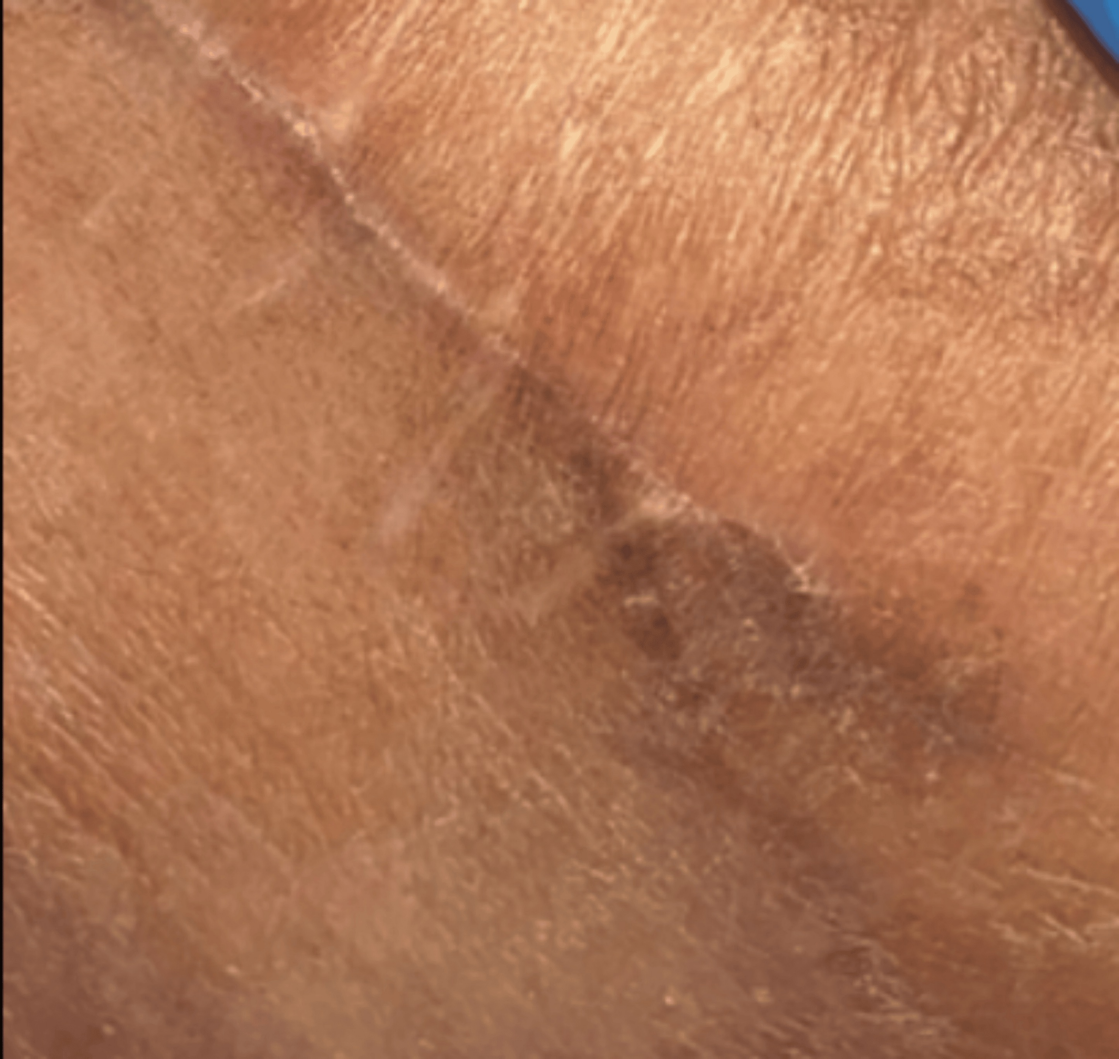
Follow-up after one month.

**Figure 9:**
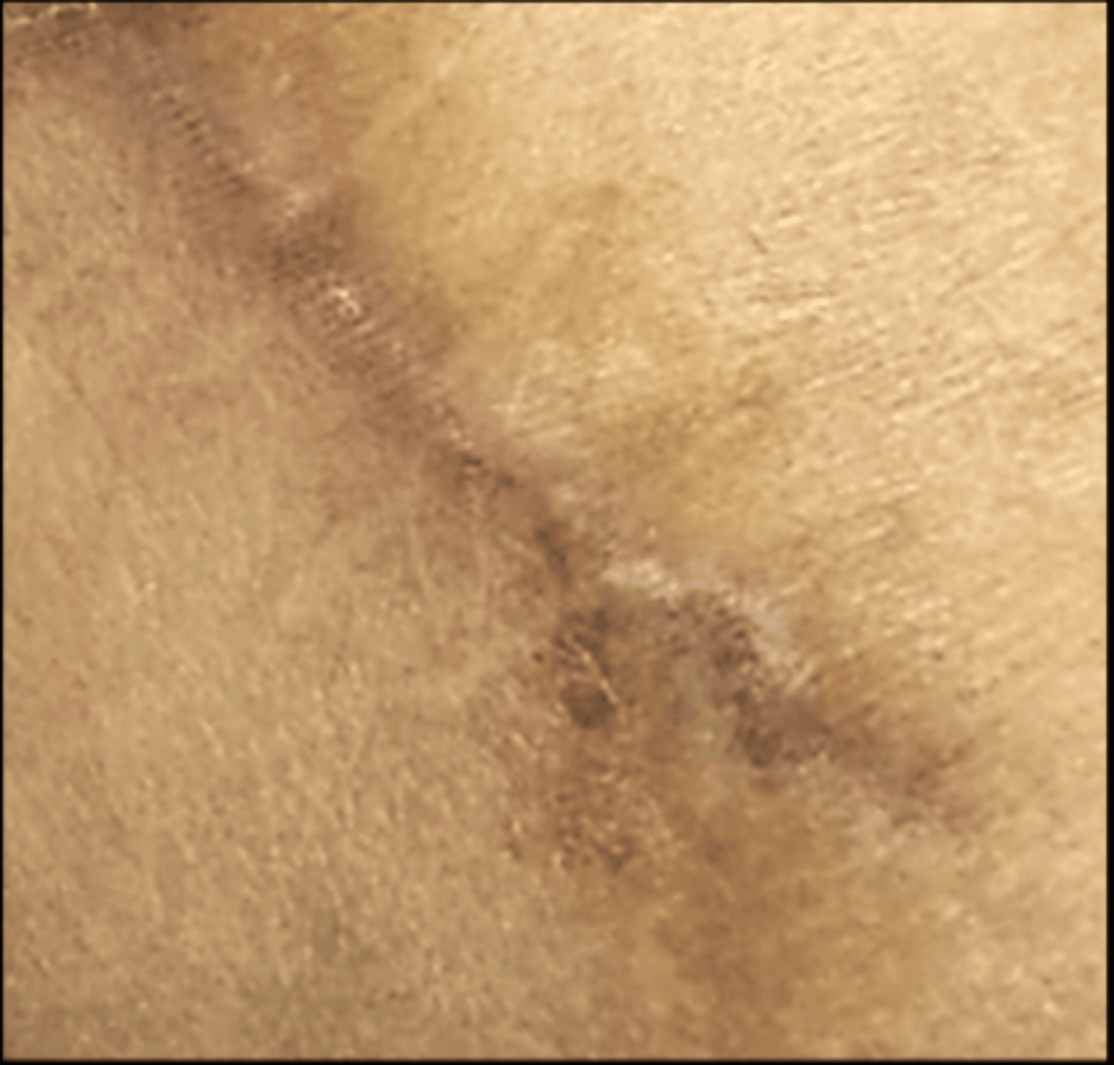
Follow-up after six months.

The patient was discharged with antibiotics, analgesics, and topical skin ointment. Histopathology confirmed a fistula tract, lined by inflamed granulation tissue along with foreign body giant cell reaction with no malignancy, which was consistent with the radiological and intraoperative findings. Skin sutures were removed on the seventh day. The patient was seen for a regular follow-up over six months (Figures 8, 9). The fistula tract healed satisfactorily, and the patient did not complain of recurrence.

## Discussion

Harvesting of autogenous bone grafts from the anterior iliac crest was established as a *gold standard* for the reconstruction of maxillary and mandibular bony defects [1]. The iliac crest is considered a suitable donor site for augmentation because of its abundant cancellous bone and its osteogenic, osteoconductive, and osteoinductive properties [4]. The iliac crest is bordered by the anterior superior iliac spine (ASIS) and the posterior superior iliac spine (PSIS). Posterior to the ASIS, the iliac crest is divided into outer and inner lips, separated by an intermediate zone known as the tubercle of the iliac crest [10]. Attached to the outer lip are the tensor fasciae latae, obliquus externus abdominis, and latissimus dorsi. In addition, along its entire length, the fascia lata is attached; the obliquus internus abdominis attaches to the intermediate line [10]. To the inner lip, however, the transversus abdominis, quadratus lumborum, sacrospinalis, and iliacus muscles, along with the iliac fascia, are attached [10]. The iliacus muscle is fan-shaped and, along with the psoas major, forms the iliopsoas muscle. The iliacus muscle originates from the iliac fossa, the anterior sacroiliac ligament, and the iliolumbar ligament. It inserts into the lesser trochanter of the femur, and it is innervated by the femoral nerve [11].

The literature reports a wide range of postoperative complications at the donor site following bone graft harvest from the AIC. There are countless reasons for the wide range of results in the literature regarding the postoperative complications of AIC bone graft harvesting. These reasons may include differences in the harvesting techniques performed, the volume of bone graft harvested, or the methods used to evaluate the results [12]. A rare complication of AIC bone graft harvesting has been reported by Sangeet et al., which was an incisional hernia secondary to the iliac crest bone graft [13]. A study by Emre et al. involving 86 patients who underwent iliac bone grafting reported gait disturbance as the most common complication, occurring in 18 patients, while the least common complications were infection and iliac fracture [14]. Mazock et al. reviewed the incidence and types of complications in 34 cases following PIC bone harvest. Three minor complications and one major complication were encountered. The sole major complication was a patient with a neurosensory deficit in the distribution of the superior cluneal nerve. The three minor complications included the postoperative formation of seromas [15]. Sudhakar et al. conducted a study to evaluate the donor site morbidity of 12 patients associated with autogenous iliac crest bone grafting for reconstruction in maxillofacial surgery. There were no major postoperative complications recorded. Whereas minor postoperative complications, including pain, contour defect, and walking difficulty, were encountered, which gradually resolved by the time of patient discharge [16].

Fistula formation can be a postoperative complication following some surgical procedures. In the case of iliac bone graft harvest surgery, a fistula can form between the bone graft donor site and another organ or tissue, such as the bladder, bowel, or skin [9]. Formation of a fistula is a rare but serious complication that can occur after iliac crest bone graft harvest surgery. In addition, a review of the literature did not reveal any studies that specifically investigated the risk of fistula formation as a postoperative complication of AIC bone graft harvesting surgery. In this case report, a fistula at the site of the left anterior iliac crest bone graft was diagnosed as a postoperative complication based on history and clinical examination. A contrasted computed tomography helped determine the origin and the extent of the fistula. However, several studies have investigated the risk of other complications, such as infection and nerve damage. These studies have shown that the risk of these complications is increased by factors such as larger bone graft size, open harvesting technique, and preexisting medical conditions [17]. Fistula formation may also be associated with these factors, but more research is needed to confirm this association.

Surgical guidelines are an essential part of any surgical procedure. They provide a framework for surgical teams to follow, ensuring that each step is taken with the utmost care and precision. These guidelines cover everything from preoperative preparation to postoperative care, and they are designed to minimize the risk of complications and ensure the best possible outcome for the patient. A key guideline to consider from this case is that surgeons should always be aware of potential complications and how to manage them before commencing the operation. Thus, thorough preoperative investigations should always be conducted to prepare for any possible intraoperative or postoperative complications [18]. In our study, likely, previous scarring was not well evaluated; thus, new incisions should avoid such areas.

One common postoperative surgical complication that patients may experience is scarring. While scarring is a natural part of the healing process, excessive scarring can be a concern in the surgical field, especially if it is a result of previous surgery. Scarring from a previous surgical procedure might alter the normal anatomy of the surgical field and make it more challenging to operate [3].

In this case report, a previous abdominoplasty has been done, and the experienced surgeon should be aware of the possibility of anatomical changes. In cases where harvesting bone grafts from a donor site is needed, it is recommended to choose a non-operated site whenever possible to minimize complications. If scarring is present in a potential donor site, an alternative surgical approach or donor site is favored whenever practically achievable. A second medical opinion or consultation can be valuable in order to ensure that patients receive the best possible care and outcomes. It is always recommended for surgeons to discuss the possibility of a second opinion with their colleagues, who can assist in delivering the best results with the minimum postoperative complications patients can have.

## Conclusions

Awareness and the ability to manage any possible postoperative surgical complications should be considered before any procedure to ensure patient safety. Management for such complications requires a clear understanding of the underlying pathophysiology, astute assessment skills, knowledge of management options, and competent technical skills. A comprehensive and effective interdisciplinary approach is required to reduce complications and achieve an optimal closure. Our report will provide valuable information about fistula formation after iliac bone graft harvest surgery. This information can be used to help surgeons expand their knowledge regarding the management of this complication when encountered.
